# Membrane “potential-omics”: toward voltage imaging at the cell population level in roots of living plants

**DOI:** 10.3389/fpls.2013.00311

**Published:** 2013-08-06

**Authors:** Antonius J. M. Matzke, Marjori Matzke

**Affiliations:** Institute of Plant and Microbial Biology, Academia SinicaTaipei, Taiwan

**Keywords:** *Ciona intestinalis* voltage sensor-containing phosphatase, nuclear electrophysiology, nuclear membranes, root cell, systems biology, roots, transmembrane potential, voltage-sensitive fluorescent protein

## Abstract

Genetically encoded voltage-sensitive fluorescent proteins (VSFPs) are being used in neurobiology as non-invasive tools to study synchronous electrical activities in specific groups of nerve cells. Here we discuss our efforts to adapt this “light-based electrophysiology” for use in plant systems. We describe the production of transgenic plants engineered to express different versions of VSFPs that are targeted to the plasma membrane and internal membranes of root cells. The aim is to optically record concurrent changes in plasma membrane potential in populations of cells and at multiple membrane systems within single cells in response to various stimuli in living plants. Such coordinated electrical changes may globally orchestrate cell behavior to elicit successful reactions of the root as a whole to varying and unpredictable environments. Findings from membrane “potential-omics” can eventually be fused with data sets from other “omics” approaches to forge the integrated and comprehensive understanding that underpins the concept of systems biology.

## INTRODUCTION

Systems biology aims to integrate multiple, large-scale “omics” data sets to create a holistic understanding of the functional principles and dynamics of complex biological systems (Zhang et al., 2010). Plant scientists are using many “omics” platforms, such as transcriptomics, proteomics, and metabolomics, to formulate systems-level descriptions of living plants at different developmental stages and under a variety of environmental conditions (Fukushima et al., 2009; Heyndrickx and Vandepoele, 2012; Keurentjes et al., 2013; Kleessen et al., 2013). Although not yet considered among “omics” approaches, the transmembrane electrical potential is an essential and universal feature of living cells and hence must be considered in any multi-scale representation of the living state (Noble, 2013). Simultaneous monitoring of membrane potential changes in populations of cells would provide a quantifiable characteristic to evaluate together with global changes in gene expression, protein abundances, and metabolite levels in systems biology research. Measurement of membrane potential has not traditionally been amenable to high-throughput analysis but recent technical advances are bringing this possibility closer to reality.

In this article, we describe progress toward adapting a technology, used originally on animal nerve cells, to record simultaneous changes in membrane potential in populations of root cells in living *Arabidopsis thaliana* (*Arabidopsis*) seedlings. The method relies on genome-encoded voltage-sensitive fluorescent proteins (VSFPs), which are able to undergo voltage-induced changes in fluorescence. In principle, VSFPs allow optical imaging of changes in membrane potential in single cells, layers of cells, and whole organisms (Peterka et al., 2011; Perron et al., 2012), thus bringing electrophysiological monitoring into the realm of systems biology. We describe the production of transgenic plants expressing various types of VSFPs targeted to different membranes in cells of roots, which are non-photosynthetic and have a low background fluorescence compared to most other plant organs. We discuss the possibilities and challenges of using genetically encoded voltage indicators in plants to study coordinated changes of the plasma membrane potential in cell populations and at multiple membrane systems within single cells.

## VOLTAGE-SENSITIVE FLUORESCENT PROTEINS

Classical methods for recording membrane potentials have generally relied on invasive tools such as microelectrodes. Motivated by the desire to carry out functional analyses of selected groups of nerve cells in the brain, neurophysiologists have been developing over the last decade experimental methods for non-invasive and synchronous monitoring of electrical activity from populations of neurons. Foremost among the tools arising from these efforts are VSFPs, which can be stably expressed in specific cell types determined by the promoter used to drive transcription and localized to distinct subcellular compartments by using appropriate targeting signals (Mutoh and Knoepfel, 2013). The technology is steadily improving and the potential for VSFPs to offer fast and sensitive optical monitoring of electrical activity in cells of living organisms led to their being declared a “Method to Watch” by Nature Methods in January 2012 (Pastrana, 2012).

The basic idea behind VSFPs is to fuse a voltage-sensing domain of a membrane protein to a single fluorescent protein or a tandem pair of fluorescent proteins and use, respectively, either changes in fluorescence intensity or FRET (Förster resonance energy transfer) to report shifts in membrane potential (Perron et al., 2009a; Mutoh and Knoepfel, 2013). The voltage-sensing domain used in the latest versions of VSFPs is derived from the voltage sensor-containing phosphatase of the sea squirt *Ciona intestinalis* (Ci-VSP; Murata et al., 2005). The transmembrane motifs S1 to S4 of Ci-VSP are thought to coordinately operate as the voltage-sensing domain. A history of the development of VSFPs is available in recent reviews (Perron et al., 2012; Mutoh and Knoepfel, 2013).

## CONSTRUCTS FOR EXPRESSING VSFPs IN PLANTS

**FIGURE 1:**
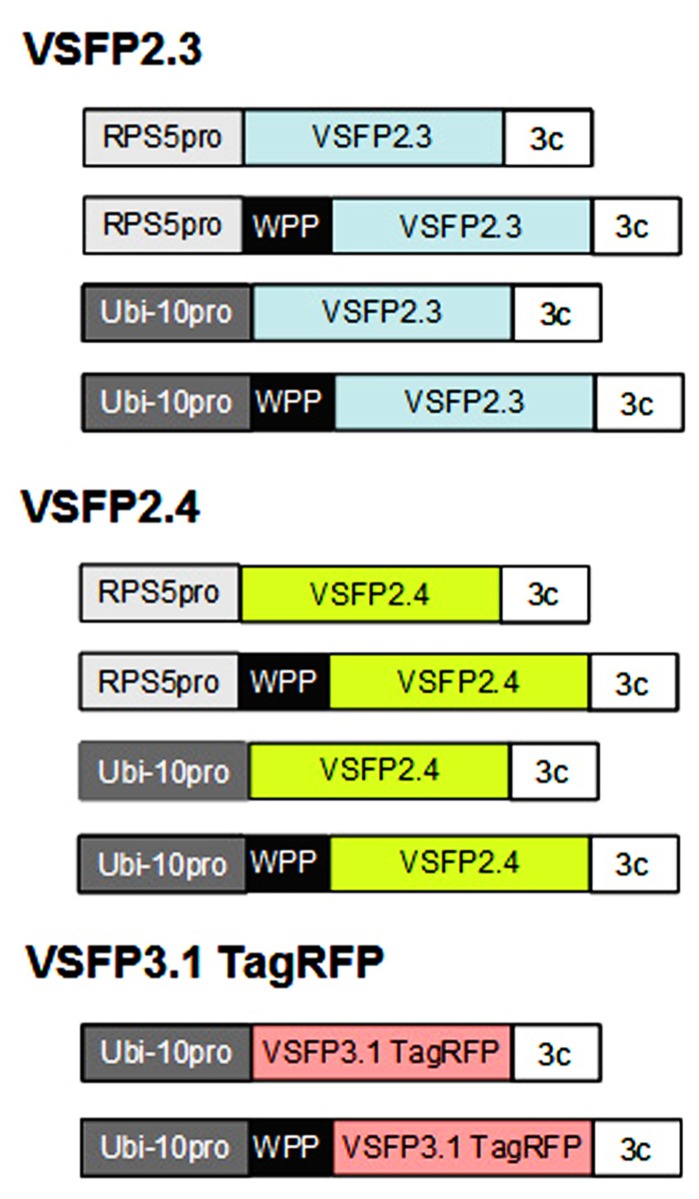
**Constructs for expressing VSFPs in plants.** Genes encoding voltage-sensitive fluorescent proteins VSFP2.3, VSFP2.4 (color of donor chromophore shown), and VSFP3.1 TagRFP (**Figure 2**) were placed under the control of either the *Arabidopsis* RPS5 promoter (RPS5pro; Weijers et al., 2001) or ubiquitin-10 promoter (Ubi-10 pro; Grefen et al., 2010), both of which are active in root cells, and the 3C transcriptional terminator from the pea rbcS3C gene (Benfey et al., 1989). Constructs were also made that included a WPP domain containing a localization signal for the outer nuclear membrane (Deal and Henikoff, 2011). The constructs were introduced into *Arabidopsis* plants using standard techniques. Around 10–20 transformed lines were obtained with each construct and screened for single locus insertions. Only lines that displayed stable and long-term expression of VSFPs in roots were retained. Images from representative lines are shown in **Figures 3,4,5**, and **7**. Constructs not drawn to scale.

**FIGURE 2:**
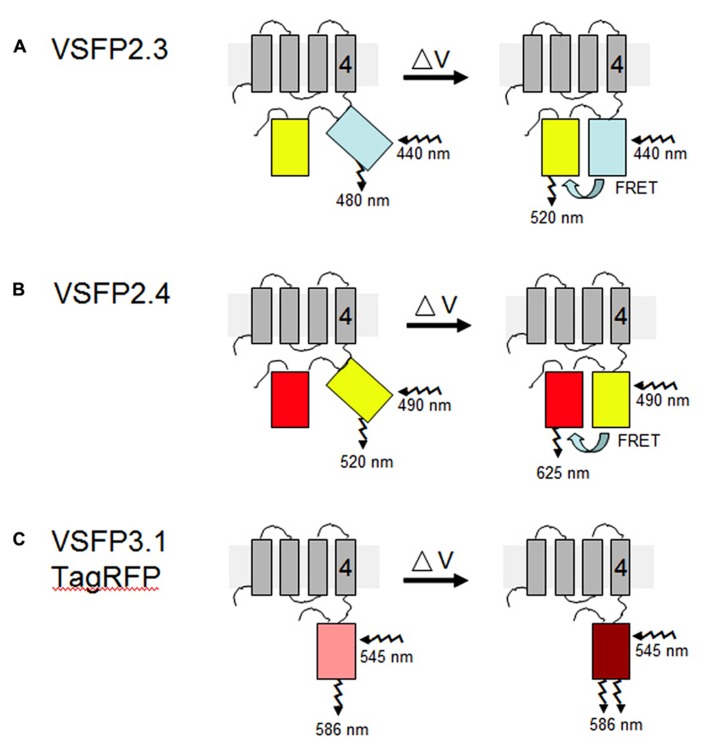
**VSFP probes of membrane potential.** Different variants of VSFPs contain a combination of a voltage-sensing domain and either a pair of donor and acceptor fluorescent proteins for FRET-based monitoring or a single fluorescent protein for intensity-based recording. One fluorescent protein is attached to the fourth transmembrane segment (gray rectangles, nr. 4) of the *C. intestinalis* voltage sensor-containing phosphatase (Ci-VSP), which comprises transmembrane motifs S1 to S4 that coordinately operate as the voltage-sensing domain. **(A,B)** In the two FRET-based systems, VSFP2.3 and VSFP2.4, illumination with the excitation wavelength of the donor protein results in fluorescence primarily at the donor wavelength under resting conditions (left). A change in the membrane potential (δV) allows energy transfer between the two fluorescent proteins, perhaps by aligning them more favorably, such that increased fluorescence of the acceptor protein is observed concomitantly with decreased fluorescence of the donor protein (right). In VSFP2.3, the donor and acceptor fluorescent proteins are mCerulean and citrine, respectively. In VSFP2.4, the donor and acceptor fluorescent proteins are mCitrine and mKate2, respectively (Akemann et al., 2010). The approximate excitation and emission wavelengths of these proteins are shown. **(C)** In the intensity-based probe, VSFP3.1TagRFP, a decrease in membrane potential leads to an enhancement of fluorescence intensity from the fluorescent protein, in this case monomeric TagRFP. The figure is based on previously published ones (Perron et al., 2012; Mutoh and Knoepfel, 2013).

We have assembled constructs for expression of three different VSFPs for use in plants (**Figure 1**). Two of these are from the second generation of VSFPs (VSFP2) and monitor changes in membrane potential through a FRET-based mechanism using a pair of fluorescent proteins. The third is derived from third generation VSFPs (VSFP3) and is based on voltage-dependent alterations in fluorescence intensity of a single fluorescent protein (**Figure 2**; Perron et al., 2009a, 2012; Mutoh and Knoepfel, 2013). The VSFP constructs were obtained from their developer, Thomas Knoepfel, and as described below, introduced into *Arabidopsis* plants under the control of various plant promoters and different subcellular localization signals. Careful selection of transcriptional regulatory signals is important because expression must be sufficiently strong to detect fluorescent signals but not so high as to promote aggregation of the fluorescent fusion proteins or interfere with membrane localization (Mutoh et al., 2011; Perron et al., 2012).

### FRET-BASED VSFPs

The FRET-based mechanism of VSFP2 proteins depends on voltage-dependent alterations in protein conformation such that energy transfer between the two chromophores is reversibly modulated by changes of membrane voltage (**Figure 2**). The two VSFP2 probes we have adapted for plants each contain a different pair of donor and acceptor fluorescent proteins fused with the Ci-VSP (Akemann et al., 2010). VSFP2.3 contains cyan-emitting fluorescent protein (monomeric) mCerulean and the yellow-emitting fluorescent protein citrine as donor and acceptor, respectively. VSFP2.4 contains (monomeric) mCitrine as a donor and mKate2, a monomeric far-red-emitting fluorescent protein (Shcherbo et al., 2009), as acceptor (**Figure 2**). Experiments with neurons have demonstrated the usefulness of testing different pairs of fluorescent protein because the species of fluorescent protein can influence the efficiency of membrane deposition and signal amplitude (Perron et al., 2009b; Mutoh and Knoepfel, 2013). The choice of which VSFP variant to use for a given purpose thus needs to be determined empirically and will depend on the cell type, degree of background fluorescence, and membrane to be targeted (Mutoh et al., 2011).

**FIGURE 3:**
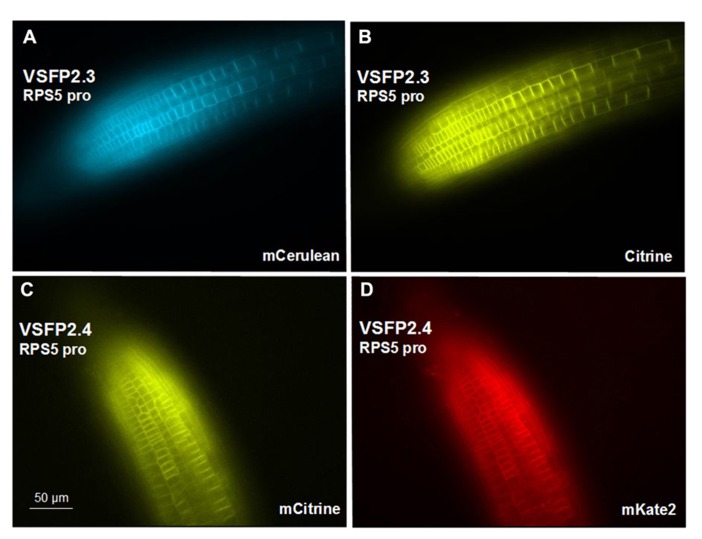
**Expression of VSFP2s from the RPS5 promoter in root tips.(A,B)** VSFP2.3, mCerulean fluorescence (left) and citrine fluorescence (right). **(C,D)** VSFP2.4, mCitrine fluorescence (left) and mKate2 fluorescence (right). Each image was made using the respective excitation and emission wavelengths for the individual chromophores (see below). Note the plasma membrane localization and the relatively uniform and equal signals from the two fluorescent proteins of each FRET pair in this region of the root. In both cases, plants are homozygous for the respective VSFP2-encoding construct. Images shown are fluorescence wide field images acquired after focusing on the top cell layers of the root. Excitation (ex) and emission (em) wavelengths (in nm) of the filter cubes used for this image: mCerulean (ex 436/em 480 nm); citrine (ex 500/em 535 nm); mKate (ex 560/em 645 nm).

**FIGURE 4:**
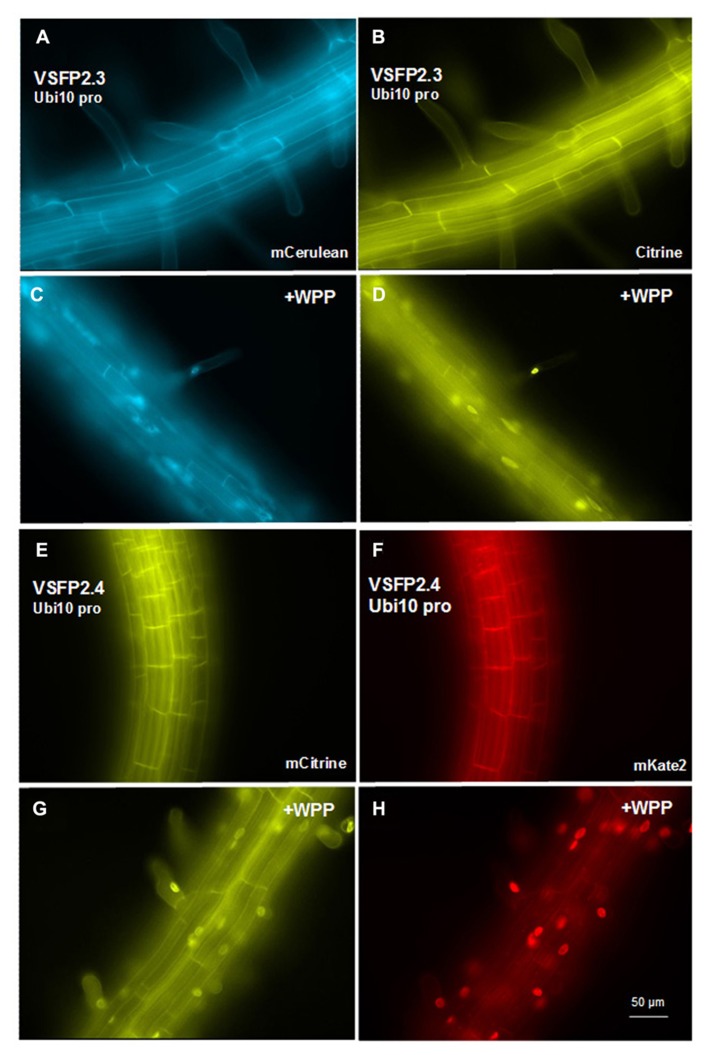
**Expression of VSFP2s from the Ubi-10 promoter and targeting to nuclear membrane in root cells.(A–D)** When driven by the ubiquitin-10 promoter, the FRET pair in VSFP2.3 (mCerulean and citrine) produces strong and uniform signals at the plasma membrane of cells in the root maturation zone **(A,B)**. When WPP is fused to the VSFP2.3 construct, fluorescence is highly concentrated at the nuclear membrane in cells of the maturation zone and in root hair cells **(C,D**, +WPP)**. (E–H)** Similar results are obtained with VSFP2.4 under the control of the ubiquitin-10 promoter (FRET pair mCitrine and mKate2; **E,F**, plasma membrane; **G,H**, +WPP, nuclear membrane). The weaker plasma membrane fluorescence than nuclear fluorescence in **(G)** and **(H)** indicates that ONM targeting of the ubiquitin-10 promoter-driven VSFP2.4 is more specific than the RPS5 promoter-driven VSFP2.4 (**Figure 7**). Images shown are fluorescence wide field images acquired after focusing on the top cell layers of the root or the root hair. Each image was made using the respective excitation and emission wavelengths of the individual chromophores (see **Figure 3** legend).

We have placed the respective constructs encoding VSFP2.3 and VSFP2.4 under the control of two distinct promoters (**Figure 1**) that display in our hands somewhat different patterns of expression in the root. The RPS5 promoter (Weijers et al., 2001) drives expression primarily in meristem region and the elongation zone (**Figure 3**). The ubiquitin-10 promoter (Grefen et al., 2010) drives lower expression in the meristem region but higher expression in the elongation and maturation zones including root hairs (**Figure 4**). Transgenic *Arabidopsis* lines homozygous for each construct have been generated and screened for the desired expression properties. In root cells, which also exhibit the aforementioned low background fluorescence, these lines display approximately equal and uniform fluorescent signals from the tandem FRET pair when imaged with the respective excitation and emission wavelengths of the individual chromophores (**Figure 3**). In mammalian cells, VSFPs based on the Ci-VSP voltage-sensing domain are efficiently targeted to the plasma membrane (Mutoh and Knoepfel, 2013) and this domain appears to work well for plasma membrane localization in root cells in the absence of a targeting signal for a specific internal membrane system (**Figures 3A–D** and **4A,B,E,F**). Long-term expression of VSFPs in the *Arabidopsis* lines we have produced does not seem to have any obvious adverse effects on plant growth, development, or reproduction.

### INTENSITY-BASED VSFPs

**FIGURE 5:**
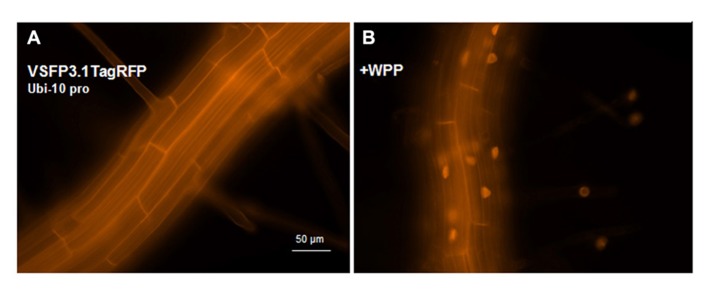
**Expression of VSFP3.1TagRFP from the Ubi-10 promoter and targeting to nuclear membrane in root cells.** Images show expression of the intensity-based VSFP3.1TagRFP from the Ubi-10 promoter at the plasma membrane **(A)** and the nuclear membrane (**B**, +WPP) in cells of the root maturation zone. In **(B)**, only some background fluorescence is seen in the plasma membrane. The four faint spots to the right in **(B)** are root hair nuclei. The excitation and emission wavelengths of TagRFP are 545 nm and 605 nm, respectively. Images shown are fluorescence wide field images acquired after focusing on the top cell layers of the root or the root hair.

VSFP3 probes are based on a single fluorescent protein that responds to voltage changes by a variation in fluorescence intensity through a mechanism that is not yet completely understood (**Figure 2**; Perron et al., 2009a,2009b,^2012^). We have adapted a VSFP3.1TagRFP construct for expression in plants by placing it under the control of the ubiquitin-10 promoter (**Figure 1**; Grefen et al., 2010). The TagRFP protein is a monomeric red fluorescent protein that is characterized by bright fluorescence, prolonged fluorescence lifetime, high pH stability and reduced tendency to form oligomers (Merzlyak et al., 2007). In an *Arabidopsis* line transformed with the VSFP3.1TagRFP construct, fluorescence signals are localized to the plasma membrane in cells of the root maturation zone (**Figure 5A**).

## TARGETING VSFPs TO THE NUCLEAR MEMBRANES

We have a long standing interest in using potential-sensitive probes to study electrical potentials at the nuclear membranes of the nuclear envelope (Matzke and Matzke, 1986,^1991^; Matzke et al., 1988,^2010^). Although the nuclear envelope is not often considered from an electrophysiological perspective, its electrical properties may influence activities in the nucleus owing to the ion transport capabilities of the nuclear membranes and the ability of electric fields to modulate DNA compaction and interactions with proteins (Musheev et al., 2010,^2013^).

**FIGURE 6:**
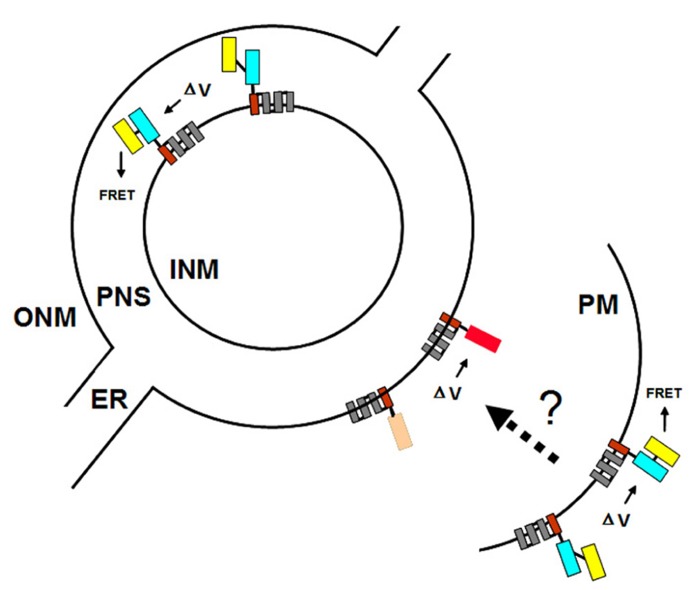
**Targeting VSFPs to different membrane systems.** The plasma membrane (PM) and the inner and outer nuclear membranes (INM and ONM, respectively) are represented by concentric black partial and full circles. For simplicity, the nuclear pores, at which the INM and ONM are fused, are omitted. The ONM is continuous with the endoplasmic reticulum (ER). The perinuclear space (PNS) between the INM and ONM is contiguous with the lumen of the ER. Targeting of VSFPs to the PM is achieved by Ci-VSP (**Figure 3**). Ideally, targeting signals specific for the INM and ONM can be identified since these two membranes may have distinct and independently regulated potentials. Using different VSFPs in the PM and ONM (or INM) would allow multicolor imaging and simultaneous monitoring of potentials at different membrane systems within a single cell. Shown here is the hypothetical situation of FRET-based VSFP2.3 in the PM (or INM) and intensity-based VSFP3.1TagRFP in the ONM. The dotted arrow and question mark indicate the possibility of detecting synchronous changes in PM and ONM (or INM) potentials using VSFPs.

The nuclear envelope of eukaryotic cells consists of two membranes, the inner and outer nuclear membranes (INM and ONM, respectively), which are fused at the nuclear pores. The nuclear pores provide the major pathway for nucleo-cytoplasmic transport of macromolecules. The compartment between the two nuclear membranes, referred to as the perinuclear space, is thought to sequester inorganic ions such as calcium for release into the nucleoplasm upon the application of appropriate stimuli (Matzke and Matzke, 1991; Charpentier and Oldroyd, 2013). Because the ONM is continuous with the endoplasmic reticulum (ER), the perinuclear space is contiguous with the lumen of the ER (**Figure 6**).

The possibility of independent control of nuclear calcium and other inorganic ions by channels and pumps in the nuclear membranes is increasingly thought to be an important contributor to the regulation of gene transcription and other processes in the nucleus (Matzke et al., 2010). Plants provide some of the best examples for independent regulation of nuclear calcium in signal transduction cascades that trigger the expression of specific genes. Several nuclear membrane-localized cation channels, CASTOR, POLLUX (Charpentier et al., 2008), and DMI (Riely et al., 2007), have been identified in forward genetic screens for nodulation-deficient mutants in legumes. These channels are essential for perinuclear calcium oscillations that precede activation of nodulation genes in response to bacterial nod factors that interact with cell surface receptors (Capoen et al., 2011).

We are interested in using nuclear membrane-localized VSFPs to monitor changes in nuclear membrane potential that occur in response to various triggering events. Such changes could be independent of the plasma membrane potential but they may also reflect synchronous changes in plasma membrane and nuclear membrane potentials following a given stimulus at the cell surface (**Figure 6**). Conceivably, such an electrically based signal transduction pathway that couples changes in plasma membrane potential to changes at the nuclear membrane could provoke rapid alterations in gene expression or influence other reactions in the nucleus (Matzke and Matzke, 1991; Shemer et al., 2008).

Given the possibility that the two nuclear membranes maintain distinct membrane potentials that are functionally significant (Matzke et al., 2010), the ideal situation would be to target one VSFP exclusively to either the INM or the ONM and a second VSFP containing a different fluorescent protein or fluorescent protein pair to the plasma membrane (**Figure 6**). To target VSFPs to the ONM, we have used the WPP domain (amino acids 1–111) of the *Arabidopsis* RAN GTPase activating protein (RanGAP1; **Figure 1**), which has been reported to be necessary and sufficient for targeting to the ONM of the nuclear envelope in plants (Rose and Meier, 2001; Deal and Henikoff, 2011).

**FIGURE 7:**
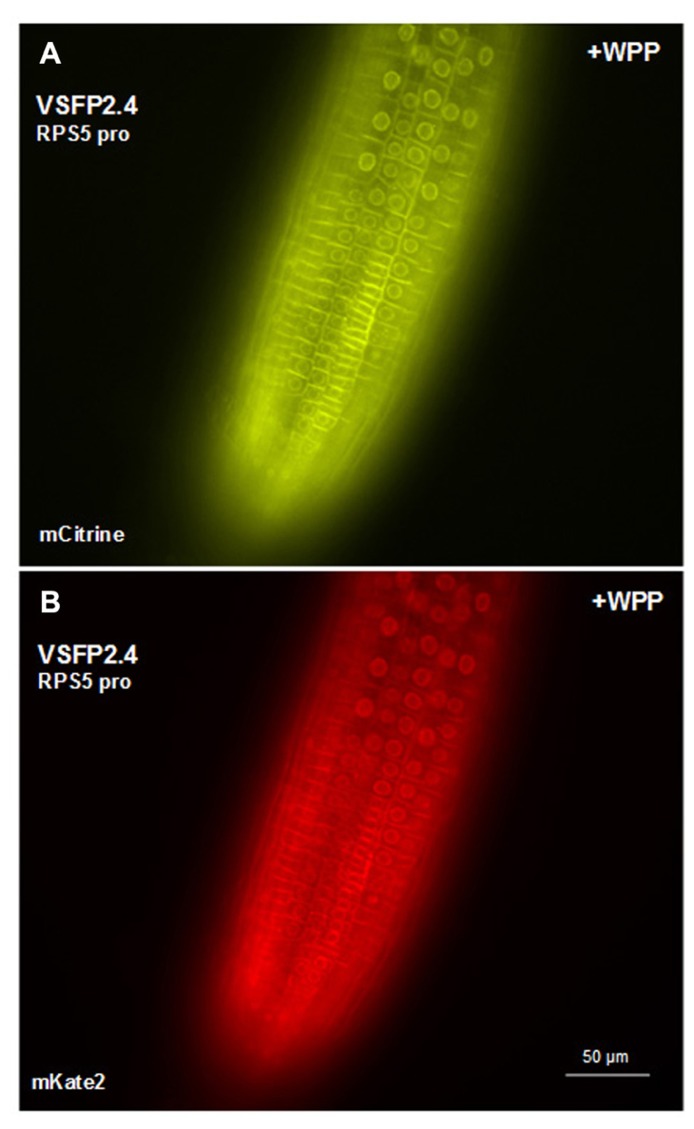
**Targeting VSFP2.4 to the outer nuclear membrane in root tip cells.** VSFP2.4 (FRET pair mCitrine and mKate) under the control of the RPS5 promoter can be targeted to the outer nuclear membrane (fluorescent circles) when coupled to the WPP domain. Considerable fluorescence is still observed at the plasma membrane, so targeting is not exclusive to the ONM. This may have something to do with the particular VSFP or cell type, since WPP localizes VSFP2.4, under the control of the ubiquitin-10 promoter, preferentially to the ONM in cells of the root maturation zone and root hairs (**Figures 4G,H**). The background autofluorescence may also vary depending on cell type at the excitation wavelength of the donor chromophore. Images shown are fluorescence wide field images acquired after focusing on nuclei in the top cell layer of the root. Each image was made using the respective excitation and emission wavelengths of the individual chromophores (see **Figure 3** legend).

In transgenic lines containing VSFP2.4_WPP under the control of the RPS5 promoter, expression of the VSFP is visible at the nuclear periphery in cells in the elongation zone and root tip but also at the plasma membrane, indicating that ONM targeting is leaky (**Figure 7**). When the ubiquitin-10 promoter was used to drive VSFP2.3_WPP expression, stronger fluorescent signals were observed at the nuclear rim than at other cell membranes in cells of the maturation zone and in root hairs (**Figures 4C,D**, compare with **Figures 4A,B**). The most specific nuclear localization of the FRET-based probes was observed with VSFP2.4_WPP under the control of the ubiquitin-10 promoter. In these plants, strong signals at the nuclear envelope were observed with little background fluorescence from other parts of the cell (**Figures 4G,H**, compare with plasma membrane staining in **Figures 4E,F**). The intensity-based probe, VSFP3.1TagRFP also displayed relatively strong ONM fluorescence when fused to WPP, with only faint plasma membrane fluorescence still visible (**Figure 5B**). The basis of the observed variations in targeting efficiency by WPP is not known but the findings illustrate that targeting VSFPs to specific membrane systems is not alwaysa straightforward matter.**A contributing factor may be differences in background autofluorescence at the distinct excitation**wavelengths of the various chromophores. Appropriate background corrections will be necessary for accurate quantitative measurements.

We have tested two localization signals for the INM, one from the mammalian protein nurim (Hofmeister and O’Hare, 2005).and a second from an *Arabidopsis* SUN protein (Graumann et al., 2010; Oda and Fukuda, 2011). However, neither of these facilitated preferential deposition of VSFPs in the nuclear envelope (data not shown). When the nuclear membrane proteome in plants is determined, it may be possible to identify more efficient targeting signals specific for the INM and ONM. We also tested an ER localization signal (Cutler et al., 2000) but for unknown reasons have been unable to obtain a reliable fluorescence signal enriched in the ER membranes.

## DETECTION

The transgenic lines we have developed are suitable for monitoring under a fluorescence microscope equipped with the proper filters and imaging software. We use a Zeiss Axioplan2 equipped with a Quad-view and MetaMorph image analysis software. For imaging, intact living seedlings expressing VSFPs can be mounted in water or buffer on a microscope slide with an indentation for leaves, covered with a 20 mm × 40 mm cover slip, and sealed with rubber cement. It is crucial to immobilize the material to prevent losing the focal plane of the membrane when adding various substances to be tested. Issues concerning signal-to-noise ratio, dynamic range, biological sensitivity, and kinetics of VSFPs have been investigated and discussed for applications in animal cells (Mutoh et al., 2011) but substantial work is still needed in these areas with respect to plant cells.

## SUMMARY

We have described the ongoing development of genetically encoded optical probes designed to record coordinated changes in electrical potential at the plasma membrane and nuclear membranes in populations of root cells in living plants. Although the development of these tools is still in the early stages, our preliminary studies and the successful use of VSFPs in animal cells are positive steps toward establishing this innovative technology in a wide range of organisms. The availability of such tools to investigate overarching electrical patterns that transcend single cell boundaries and single membrane systems will contribute needed information on a currently underappreciated dimension of plant systems biology.

## Conflict of Interest Statement

The authors declare that the research was conducted in the absence of any commercial or financial relationships that could be construed as a potential conflict of interest.
